# A hybrid reference-guided de novo assembly approach for generating *Cyclospora* mitochondrion genomes

**DOI:** 10.1186/s13099-018-0242-0

**Published:** 2018-04-10

**Authors:** G. R. Gopinath, H. N. Cinar, H. R. Murphy, M. Durigan, M. Almeria, B. D. Tall, A. J. DaSilva

**Affiliations:** Office of Applied Research and Safety Assessment (OARSA), Center for Food Safety and Applied Nutrition (CFSAN), US Food and Drug Administration, 8301 Muirkirk Road, Laurel, MD 2070 USA

**Keywords:** Genome sequencing, Mitochondrion, De novo assembly, Reference genome, Single nucleotide polymorphisms, Cyclosporiasis, Subtyping

## Abstract

*Cyclospora cayetanensis* is a coccidian parasite associated with large and complex foodborne outbreaks worldwide. Linking samples from cyclosporiasis patients during foodborne outbreaks with suspected contaminated food sources, using conventional epidemiological methods, has been a persistent challenge. To address this issue, development of new methods based on potential genomically-derived markers for strain-level identification has been a priority for the food safety research community. The absence of reference genomes to identify nucleotide and structural variants with a high degree of confidence has limited the application of using sequencing data for source tracking during outbreak investigations. In this work, we determined the quality of a high resolution, curated, public mitochondrial genome assembly to be used as a reference genome by applying bioinformatic analyses. Using this reference genome, three new mitochondrial genome assemblies were built starting with metagenomic reads generated by sequencing DNA extracted from oocysts present in stool samples from cyclosporiasis patients. Nucleotide variants were identified in the new and other publicly available genomes in comparison with the mitochondrial reference genome. A consolidated workflow, presented here, to generate new mitochondrion genomes using our reference-guided de novo assembly approach could be useful in facilitating the generation of other mitochondrion sequences, and in their application for subtyping *C. cayetanensis* strains during foodborne outbreak investigations.

## Background

*Cyclospora cayetanensis* is an important apicomplexan parasite causing cyclosporiasis, a common foodborne illness [1] worldwide. Due to the globalization of the food supply, this apicomplexan parasite is prevalent in both endemic regions producing food and non-endemic areas where food is imported [2, 3]. The lack of animal models or cell culture systems for *Cyclospora* and the limited availability of its oocysts have hampered its genomics and the development of efficient genotyping tools. With the advent of new sequencing methods, the number of genome sequences from *C. cayetanensis* is growing over the past 4 years, however, with no immediate solution to the subtyping problem. Our group ([4, 5]) and others [6, 7] have recently published genomes for the *C. cayetanensis* organelles—apicoplast and mitochondrion, and whole genome sequences [8, 9] that provide a glimpse into its biology. A few PCR targets amplified from geographically distinct strains have been tested for subtyping [10]. Unlike the case with foodborne bacteria (https://www.fda.gov/Food/FoodScienceResearch/WholeGenomeSequencingProgramWGS/), the impact of genomics on the development of molecular epidemiological methods based on genomics are not yet fully realized for *C. cayetanensis* due to the complexity of obtaining high quality genomic information from NGS datasets.

Growing amounts of genomic data from mixed DNA samples recovered from clinical, animal or environmental samples often result in assemblies and variant determinations with various levels of confidence. High-quality reference genomes are critical for quality assurance and reproducibility [11] to support assembly, annotation and accurate variant determination with NGS metagenomic datasets from uncultivable microorganisms. We previously published a reference genome for the *C. cayetanensis* apicoplast [5] which involved applying manual curation and bioinformatic analysis of in-house metagenomic sequence datasets from stool samples to re-construct 11 new apicoplast assemblies. This reference genome was used to identify 25 genomically variable regions or hotspots in the apicoplast genomes of these 11 different clinical strains. In the current work, we (a) evaluated a high-resolution, annotated mitochondrial genome KP231180 [4] and propose that it be adopted as the mitochondrial reference genome; (b) developed a hybrid, reference-guided NGS approach to build new assemblies de novo; (c) demonstrated the usefulness of this hybrid approach to generate three mitochondrial genome assemblies from metagenomic sequence datasets from cyclosporiasis clinical samples, and (d) identified alleles based on the reference-genome sequence of six new and public mitochondrion assemblies. This workflow is anticipated to generate mitochondrial genomes of comparable quality from different samples for genotyping purposes and possible source attribution.

## Methods

**Fig. 1:**
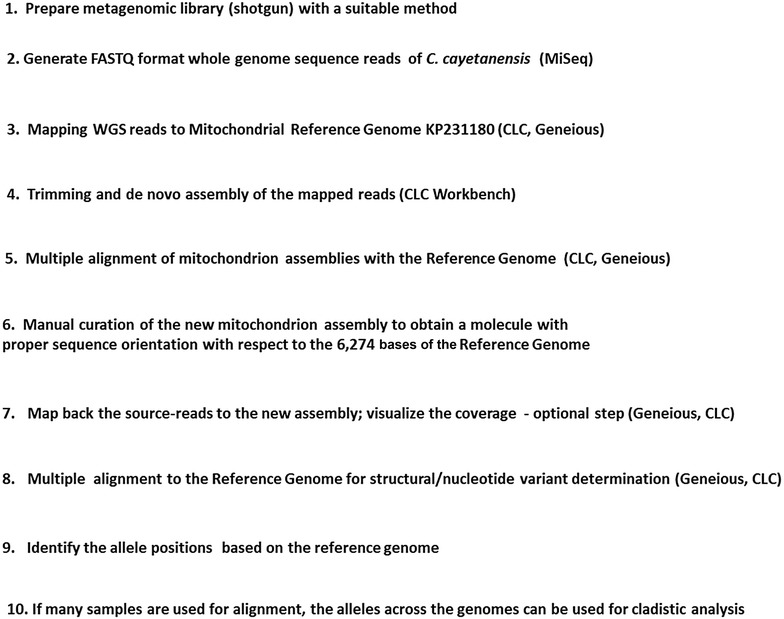
Workflow chart for recovering mitochondrial genomes from metagenomic sequence datasets. The mitochondrion reference genome KP231180 was used twice to generate new assemblies using metagenomic reads from stool samples. First, NGS reads were mapped to the reference to gather mitochondrion-specific sequences. Secondly, after the assembly the orientation of the sequences in the new assemblies were corrected to be in alignment to the reference genome for downstream analysis. It is anticipated that the reference-guided, de novo assembly workflow can be modified to fit the nature of any NGS datasets

**Fig. 2:**
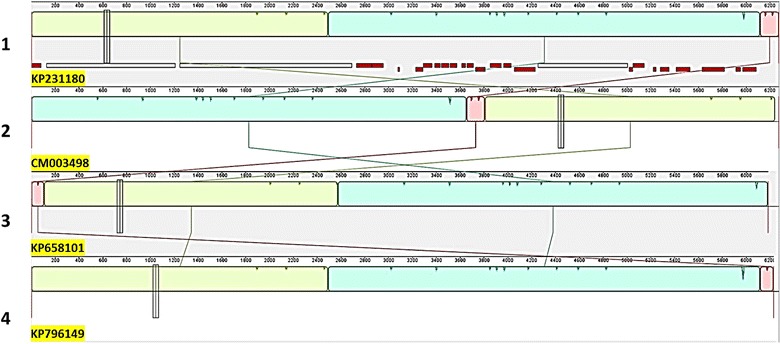
Multiple alignment of the four public mitochondrial genome assemblies. Track 1: annotated KP231180 [4]; track 2: CM0003498; track 3: KP658101 [7] and track 4: KP796149 [6]. KP231180, the longest of the four assemblies in track 1 was compared with other three mitochondrial assemblies) using the progressiveMauve algorithm implemented on the Geneious suite. CM003498 and KP658101 assemblies had shuffled sequences indicated by crisscrossing lines connecting a terminal pink block of sequence from track 1 re-located to different regions in tracks 2 and 3. Annotations could not be mapped from KP231180 to CM003498 due to the sequence shuffling. The assemblies in tracks 3 and 4, namely, KP658101 and KP796149, were also of different lengths mainly due to some deletions not seen in this illustration

**Fig. 3:**
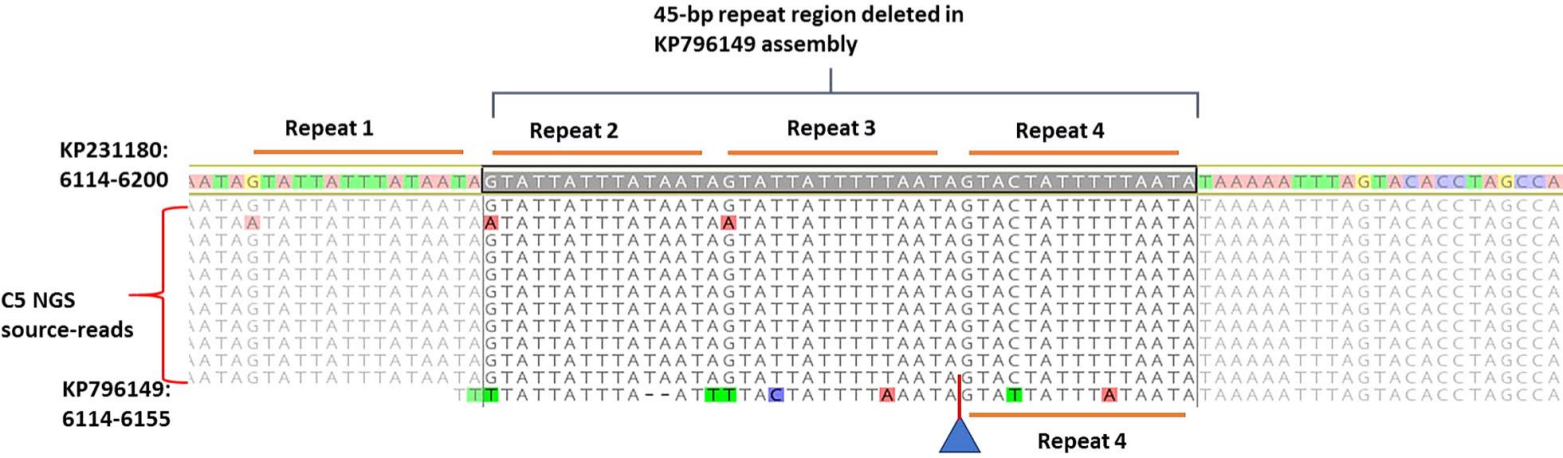
Identification of four repeats in the mitochondrion genome potentially affecting de novo assembly quality. A 45 bp deletion was observed in the terminal region of 6129 bp-long KP796149 assembly (greyed out sequence in the top track), which was investigated further. Four 15-mer repeats have been observed in the 6274 long KP231180 assemblies. Three of the four repeats were seen missing in the KP796149 assembly after alignment indicated by a blue triangle with a red bar (last track). Though the sequence from KP796149 appears to be aligned with the reads and KP231180, the colored and missing bases in the last track point to a forced misalignment. The C5 source-reads (used as a representative of all the three source-read datasets) mapped to this region in KP231180 and its assembly contained these repeats as expected (11 tracks in the middle). The presence of just one of the four repeats in KP796149 sequence [6] would result in improper mapping of the reads and render any dependent de novo assembly incomplete

Publicly available *C. cayetanensis* mitochondria sequences—KP231180 [4], HCNY (KP658101; [7], CM003498 (unknown strain) and HEN01 (KP796149; [6]—were downloaded from GenBank, NCBI (https://www.ncbi.nlm.nih.gov/) and evaluated to be used as the reference genome. Purification of oocysts from stool samples, total DNA extraction from purified oocysts, metagenomic library preparation, and genome sequencing methods were carried out as described by Cinar et al. [5]. Genomic DNA was extracted from oocysts purified from three different patient stool samples in our collection (originally obtained from Nepal including C5, C8 and C10 [NCBI Biosamples: SAMN04870148, SAMN04870149 and SAMN04934518, respectively]). Metagenomics libraries were generated (Fig. 1 workflow: *Step 1*) using the Ovation Ultralow Library System V2 (NuGen) and sequenced (*Step 2*) using an Illumina MiSeq instrument (https://www.illumina.com). C5 and C8 libraries were used for a paired-end (300 × 2 cycles) and a single-end (600 cycles) individual runs. For each sample, the reads from these two individual runs were pooled before mapping (Table 1). Reads from C10 sample were obtained from a paired-end run (300 × 2 cycles) only. The read-mapping was carried using the mapping tool in CLC Workbench 8.5 under the default conditions (https://www.qiagenbioinformatics.com/products/clc-genomics-workbench). The reference genome KP231180 was used for mapping the total metagenomic reads to collect *C. cayetanensis* mitochondrion-specific source-reads from each sample (*Step 3*) and for mapping back the source-reads to confirm the accuracy. The trimming tool of the CLC suite was used for quality filter and adaptor trimming of the source-reads from each sample. Adaptor trimming was carried out based on a list of known adaptor sequences and the quality trimming was done using the default parameters of the tool. For the de novo assembly, the contig length was set to a minimum of 500 bp while other default configurations were maintained (*Step 4*). A feature to map back the reads to the generated contig to refine the assembly was opted in the de novo tool of CLC suite. Initial C5, C8 and C10 assemblies were aligned (*Step 5*) with the reference genome and corrected (*Step 6*) using the ‘Map to Reference’ tool in Geneious tools to address sequence rearrangements. Source-reads from each of the samples were mapped to the respective assemblies to visualize the coverage using the CLC suite mapping tool. This is an optional step in the workflow to understand the extent of coverage of the assembly when using mapped reads (*Step 7*). The new assemblies were queried against the reference genome and aligned to detect any structural (InDels) or nucleotide variants (*Step 8*) using the Geneious tools. Variants in the query genomes were identified with reference to the base position in the reference genome (*Step 9*). The above steps 1–9 followed in our protocol were consolidated into a new workflow (Fig. 1) to generate de novo mitochondrial genome assemblies from NGS reads. progressiveMauve [12] implementation in Geneious (www.geneious.com) was utilized for multiple alignment and visualization of any anomalies in the genomes as seen in Fig. 2. A sequence template comprising of the concatemeric tail:head junction (1085 base pairs (bp); stretching from 6001 to 6274 and 1 to 819) from KP231180 was generated for identifying reads mapping to the junction or some repeat region seen partially in Fig. 3. The mitochondrial assemblies from C5, C8 and C10 were annotated based on the reference genome using BLAST (https://blast.ncbi.nlm.nih.gov/Blast.cgi) and BankIt utilities of NCBI submission portal [13]. For NGS reads mapping, alignment and visualization, and variant detection, appropriate tools on either Geneious suite or CLC workbench can be used interchangeably. Either set of tools were observed to yield similar results with the genomes used and generated in this study.

**Table 1:** Results of mapping, trimming and assembly of sequencing reads from three *C. cayetanensis* strains

Attribute	*C. cayetanensis* assemblies		
	C5	C8	C10
Total reads (metagenomic)	34 × 10^6^	33 × 10^6^	7.3 × 10^6^
Source-reads (untrimmed)^1^	298,214^1a^ (0.87%)	205,267^1a^ (0.62%)	38,206^1b^ (0.52%)
Source-reads (trimmed)^1^	298,214	205,265	38,206
Average read length (bp)	245	247	237
Reads used in the assembly	294,688	203,066	37,850
Reads mapped back^2^	298,144 (99.98%)	205,226 (99.98%)	38,195 (99.97%)
Genome coverage^3,4^	> 11,000 ×	> 8000 ×	> 1400 ×

^1^ Source-reads for each sample were obtained by mapping total metagenomic reads on to the KP231180 reference genome; ^1a^ for either of C5 and C8, metagenomic reads from a 300 × 2 cycles paired-end and a 600 cycles single-end runs from a single library prep were pooled before mapping; ^1b^ for C10, reads from a single 300 × 2 paired end run were used for mapping

^2^ Source-reads from each sample were mapped back to the reference genome and corresponding assembly. The same percentage was observed in either instance for each sample

^3^ Genome coverage (assuming all reads are from single-end runs) was based on the TechNote from Illumina available at https://www.illumina.com/documents/products/…/technote_coverage_calculation.pdf

^4^ For each sample, a mitochondrion assembly comprising of a single 6274 bp long contig was generated following the workflow described in Fig. 1. This was used to calculate the coverage

## Results and discussion

### Determination of the reference genome for *C. cayetanensis* mitochondrion

**Fig. 4:**
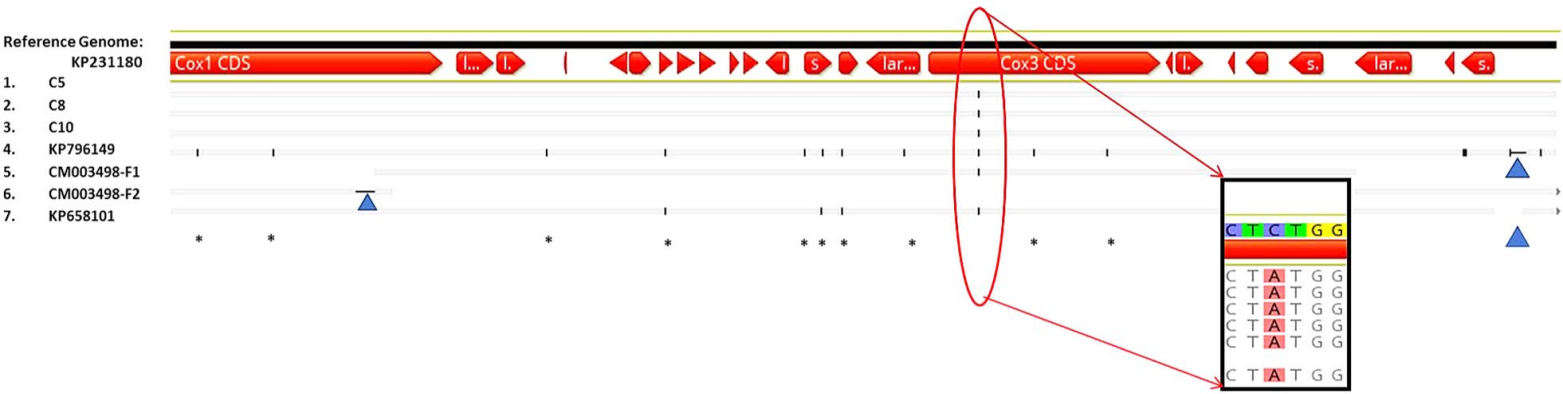
Comparison of the new mitochondrial assemblies and the public sequences with the reference genome. The topmost track with CDS (annotations in red) is the reference genome, KP231180. Tracks 1–3: 6274 bp long C5, C8 and C10 assemblies respectively; tracks 4: 6229 bp long KP796149 [6] assembly; track 5–6: 6273 bp long CM003498 assembly split into two fragments by the alignment program to obtain collinearity. The fragment start-stop positions are given in the track name; track 7: 6184 bases long KP658101 [7] assembly. The three mitochondrion assemblies CM0003498, KP658101 and KP796149 along with the new C5, C8 and C10 sequences were compared with the reference genome evaluated in this study. The mapping and visualization were carried out using Geneious suite utilities to highlight SNPs. A synonymous transversion mutation was identified in all six assemblies in comparison with the reference. The alleles present in the query genomes with respect to the base in position 4415 of the reference genome are shown in the inset box. In addition, anomalous SNPs (marked by *) were observed in KP796149 and KP658101. The sequence integrity appears to be affected in the public sequences due to sequence shuffling (tracks 5–6 of CM0003498) and deletions indicated by blue triangles (tracks 4–7 for three genomes). The manually curated KP231180 [4] was used as the reference genome (topmost track with CDS annotations) for further analysis to avoid potential mis-assembly and propagation of false positive variants possible with other three genomes

We evaluated four publicly available mitochondrial genomes of varying lengths [KP658101 (6184 bp), CM003498 (6273 bp), KP796149 (6229 bp) and KP231180 (6274 bp)] to be considered as a reference genome for future comparative sequence analysis. When aligned to the longest molecule KP231180, the three other genomes had deletions and rearrangements within their sequences (Figs. 2, 3 and 4). Multiple alignments of these four public mitochondrial sequences revealed shuffling of sequence blocks in KP658101 (Fig. 2) and CM003498 (Fig. 4, tracks 5 and 6). These anomalies likely arose due to the use of different library preparation and assembly protocols and may impact their utility as reference genomes. For example, the shuffled terminal region of KP658101 (track 3, Fig. 2, pink block) in comparison to other assemblies may result in improper annotation of the assembly. The missing 90-bp region of KP658101 (indicated by blue triangle, Fig. 4, track 7) may result in artifacts if used for generating new assemblies. In the case of CM003498, a sequence mis-assembly (track 2, Fig. 2, pink block in the middle) was identified from this alignment. This assembly was split into two fragments during multiple alignment analyses (illustrated as two tracks in Fig. 4, tracks 5 and 6) as a consequence of this sequence shuffling. Also, a small region was seen deleted in this molecule (blue triangle in track 6, Fig. 4). GenBank annotation was not available for both of these sequences. KP796149 [6] contained a 45 bp-deletion in the terminal repeat region of the molecule (Fig. 3). Of the four 15-mer repeats in this region found in the reference KP231180 (Fig. 3; four red lines tagged ‘Repeats 1-4′ in the top track), only one was identified in KP796149 (Fig. 3, red horizontal lines in the bottom track). These deletions in the repeat regions potentially limit the choice of KP796149 to be considered as a reference genome. In addition, both KP796149 and KP658101 contained an unusual number of alleles (positions marked by ‘*’ in Fig. 4, tracks 4 and 7) not seen in other strains. In contrast to these three sequences, KP231180 assembly and structure were independently verified by Sanger sequencing of genome-spanning amplicons, NGS-based reads datasets and comparative genomics as part of the manual curation process described by Cinar et al. [4]. To avoid potential issues arising from shuffling and deletions seen in the other three genomes, the high resolution and annotated KP231180 genome was designated as the mitochondrial reference genome and was used to assemble mitochondrion genomes from other strains in this study.

### Generation of three new genome assemblies from metagenomic sequence datasets

Three new mitochondria genomes were assembled using the mitochondrial reference genome KP231180 as outlined in the workflow (Fig. 1). The reference genome-based approach described in this study was developed over the years specifically to address the limited amount and metagenomic nature of *C. cayetanensis* DNA, and resembles a reference-guided assembly method recently described for single genomes [14]. The mitochondrion assembly KP231180 [4] and a dozen apicoplast assemblies from *C. cayetanensis* [5] were generated using this reference-guided workflow. A total of 298,217, 205,265 and 38,206 source-reads (less than 1% in each of the three samples) were first obtained from the large metagenomic sequence datasets of C5, C8 and C10 strains respectively (Table 1) by mapping to the mitochondrial reference genome. The source-reads were trimmed and assembled using CLC genome Workbench as described (Table 1). For each strain, the trimmed source-reads were used in the CLC Workbench de novo assembly tool to generate a single, contiguous contig. When compared with the reference genome in the Geneious suite (data not shown), the linear contigs of the three initial assemblies started from different base positions due to randomly-oriented assembling processes. *C. cayetanensis* mitochondrial genomes are known to be concatemeric [4, 6] wherein the tail region of one mitochondrial genome fuses with the head region of the second molecule creating a native tail:head junction. Sequencing reads overlapping this junction may possibly lead to mis-assembly. The lack of collinearity between the reference genome and these new assemblies also impaired the ability to align them for base-level comparisons. Alignment programs like Mugsy and Mauve [15] allow artificial re-arrangements of sequences to create locally collinear blocks (LCBs) for alignment. In our reference-guided approach, we added a manual curation step in which the reference genome KP231180 was used to correct any randomly-oriented contig to form a collinear assembly that could be readily aligned with other genomes end-to-end. In this step, blocks of sequences in each genome were manually rearranged to achieve synteny with the reference genome. This corrected assembly could be used in multiple alignments for detecting any relevant single nucleotide polymorphisms (SNPs) and/or InDels.

Comparison and correction with the reference genome resulted in a 6274 bp-long assembly for each of the three strains, C5, C8 and C10. A very high coverage for each genome was achieved suggesting the utility of NGS in obtaining a good quality genome for this small organelle from different patient stool samples (Table 1). The corrected assemblies (Fig. 4, tracks 1–3) of the three strains aligned to the reference genome without any shift in sequences or mis-alignment. When the source-reads for each sample were mapped back to the respective mitochondrion genome to detect and eliminate any spurious insertions/deletions and misassembled sequence regions, no valid gaps were detected. The source-reads mapped from each sample mapped back to the respective assembly or the reference genome with up to 99.97% of the reads (Table 1) suggesting a very high degree of homology between the reference and new genomes. DNA repeat sequences are known to create errors in alignment and assembly particularly with NGS datasets [16]. To examine whether the known repeats of *C. cayetanensis* mitochondrial genome interfered with the mapping efficiency, the 1085 bases long fragment of the reference genome sequence (described earlier in the “Methods” section) was challenged with NGS reads from the three samples. As illustrated with the C5 data set as a representative sample in Fig. 3, source-reads from the three samples mapped without any gaps to the repeat-rich region of the target shown (from 6114 to 6200 spanning four repeat blocks), highlighting the quality of recovered reads using this reference-guided approach. The annotation based on the reference genome resulted in identifying three protein coding genes (*cytB, cox1 and cox3*), in addition to 14 LSU and nine SSU fragmented rRNA genes in each of the genomes as expected and suggesting that an intact assembly structure was obtained. When the 1085 bp template containing only the concatemeric junction (tail:head) of KP231180 was interrogated with source-reads from the sample datasets, numerous read-throughs from this region were observed (data not shown). The tail:head junction originally reported by Cinar et al. [4] in the reference genome and in the strain HEN01 by Tang et al. [6], was also seen in each of the three strains used in this study, confirming the native, concatemeric structure of the *Cyclospora* mitochondrion genome. All these results provide evidence that point to a high rate of specificity and accuracy of the mapping and the assembly processes, resulting in de novo mitochondrial assemblies retaining structural integrity similar to the reference genome. It has to be noted here that there are reference-based assembly methods available to circumvent the de novo step used in this study. For example, Cinar et al. [5] described a modification of this workflow in which after mapping reads to a reference genome, Geneious tool allowed the extraction of a linear consensus sequence formed by assembling overlapping reads. A contiguous new assembly was generated as a result of significantly greater depth of sequencing thus avoiding gaps and the formation of multiple contigs. Tools like AlignGraph [17] extend the use of paired-end sequencing reads to map to a closely related reference genome and creating contiguous or scaffolded assembly. Schneeberger et al. [18] used a hybrid approach similar to our method by combining reference-guided assembly with a de novo assembly. Such a hybrid approach was efficient in identifying new sequence and structural differences among the strains as observed also in the case of *C. cayetanensis* apicoplasts [5].

### Sequence comparison and variant calling based on the reference genome

The mitochondrial genomes from the three strains C5, C8 and C10, and three other publicly available genomes were aligned with the reference genome to identify alleles among them. Based on this comparison, a synonymous, C- > A transversion was found for position 4415 (*cox3* gene) on the reference Genome KP231880 in all the six strains used in the comparison (red oval zoomed into the inset box, Fig. 4). Specific reads mapping to this region confirmed the accuracy of this allele identification (data not shown). KP796149 contained seven unique alleles and shared three more alleles with KP658101 (Fig. 4, track 7, marked by ‘*’), which need to be independently verified in a larger number of *C. cayetanensis* strains. It is interesting to note that in the Nepal samples C5, C8 and C10, the 34 kb apicoplast genomes were indistinguishable from each other [5] as were their 6.7 kb mitochondrion genomes (Fig. 4; tracks 1–3). It has been observed in foodborne bacteria [19, 20] and in other apicomplexans [21–23] that strains from the same geographical locations display different levels of differentiation in their variant markers, a genomic feature that could be applied in creating identification/barcoding schemes for subtyping or strain-level identification.

### Significance of the current work

We are presenting a hybrid reference-guided, de novo genome assembly approach for *Cyclospora* mitochondrion genomes. As part of this study, we described a *C. cayetanensis* mitochondrial reference genome that could be routinely used in building new mitochondrial assemblies, and for genome comparisons. This robust approach yielded three new mitochondrial assemblies derived from metagenomic sequencing data useful in variant determination with high confidence when aligned with the mitochondrial reference genome. This study complements a similar reference genome-based workflow for obtaining high quality *Cyclospora* apicoplast genomes, first reported by our group in Cinar et al. [5]. Our reference guided workflow, enunciated from our work on the NGS datasets from stool samples, should be instrumental for the addition of more *C. cayetanensis* mitochondrial genome data from food or environmental samples. A standard and routine workflow for the extraction and recovery of mitochondrial sequences from contaminated clinical, food and environmental samples would foster the identification of potential subtyping markers for source-tracking and in the development of molecular diagnostic detection tools for *C. cayetanensis* to assist with outbreaks investigations.

The newly assembled mitochondrial genomes from *C. cayetanensis* strains C5, C8 and C10 are available (Accessions MG831586, MG831587 and MG831588 respectively) from NCBI Bioproject: PRJNA357478 *C. cayetanensis* Mitochondrial genome sequencing for Molecular Serotyping, a component of FDA *Cyclospora* GenomeTrakr (Bioproject PRJNA357477).

## Abbreviations

NGS: next generation sequencing
WGS: whole genome sequencing
bp: base pairs
kb: kilobase
SNPs: single nucleotide polymorphisms
